# Reduced neural representation of arm/hand actions in the medial posterior parietal cortex

**DOI:** 10.1038/s41598-018-37302-2

**Published:** 2019-01-30

**Authors:** A. Bosco, R. Breveglieri, M. Filippini, C. Galletti, P. Fattori

**Affiliations:** Department of Biomedical and Neuromotor Sciences, University of Bologna, Bologna, Italy

## Abstract

Several investigations at a single-cell level demonstrated that the medial posterior parietal area V6A is involved in encoding reaching and grasping actions in different visual conditions. Here, we looked for a “low-dimensional” representation of these encoding processes by studying macaque V6A neurons tested in three different tasks with a dimensionality reduction technique, the demixed principal component analysis (dPCA), which is very suitable for neuroprosthetics readout. We compared neural activity in reaching and grasping tasks by highlighting the portions of population variance involved in the encoding of visual information, target position, wrist orientation and grip type. The weight of visual information and task parameters in the encoding process was dependent on the task. We found that the distribution of variance captured by visual information in the three tasks did not differ significantly among the tasks, whereas the variance captured by target position and grip type parameters were significantly higher with respect to that captured by wrist orientation regardless of the number of conditions considered in each task. These results suggest a different use of relevant information according to the type of planned and executed action. This study shows a simplified picture of encoding that describes how V6A processes relevant information for action planning and execution.

## Introduction

Area V6A is a high-order cortical region of the medial posterior parietal cortex^1^ involved in reaching and grasping movements in both monkeys^1–3^ and humans^4–7^. Spatial location, shape and orientation of objects, that are the basic kinds of information for a correct execution of hand movements, are also highly influential in V6A^8–11^. Additionally, the activity related to reaching and grasping of V6A neurons is modulated by both the visual information from the environment and from the arm-target interactions^12–14^. The common approach of previous studies on V6A was to correlate single external parameters, such as visual information, target location, orientation and shape, with the neural activity. However, it is well known that neural responses of single neurons encode multiple functions^15–19^, and most neurons encode several task parameters simultaneously generating “mixed” representations^20^ that ignore how exactly each of these parameters is represented at the neural level^21^. This encoding process can be studied by dimensionality reduction methods, that automatically summarize all the important features of the population activity in a single figure, such as the demixed principal component analysis (dPCA)^22^.

Here we present a population study with data from previous studies from our laboratory^12–14^ in which V6A neurons were tested while the animal executed reaching and grasping movements in light and dark conditions in three different tasks: the *reach direction* task, in which we investigated the effect of visual information and target location on the neural responses of V6A population; the *wrist orientation* task, in which we assessed the effect of visual information and wrist orientation; the *grip type* task, in which we evaluated the effect of visual information and grip type. In the present work, we wanted to assess the influence of all these task parameters on the population activity of V6A to obtain a population picture of the encoding process free from noise and redundant information. We also analysed how V6A neurons discriminate these parameters across task progression in the three different tasks. We have found the relative percentage of population variance related to visual information, target location, wrist orientation and grip type and how they were discriminated across the time depended on the task requirements suggesting a flexible encoding of variables not visible with single-cell analysis. This analysis tool allowed us to obtain a simplified snapshot of V6A functional properties and to observe the population encoding without previous selection of neural activity. The output of such a type of analysis results very suitable for neuroprostethics readout.

## Results

### Contribution of visual information, spatial position, wrist orientation, and grip type to reaching and grasping activity in V6A

Three different populations were employed to investigate the neural pattern of discharge when macaque monkeys performed reaching and grasping actions. The statistical analyses were carried out on these cell populations without any pre-selection of neural activity.

**Figure 1:**
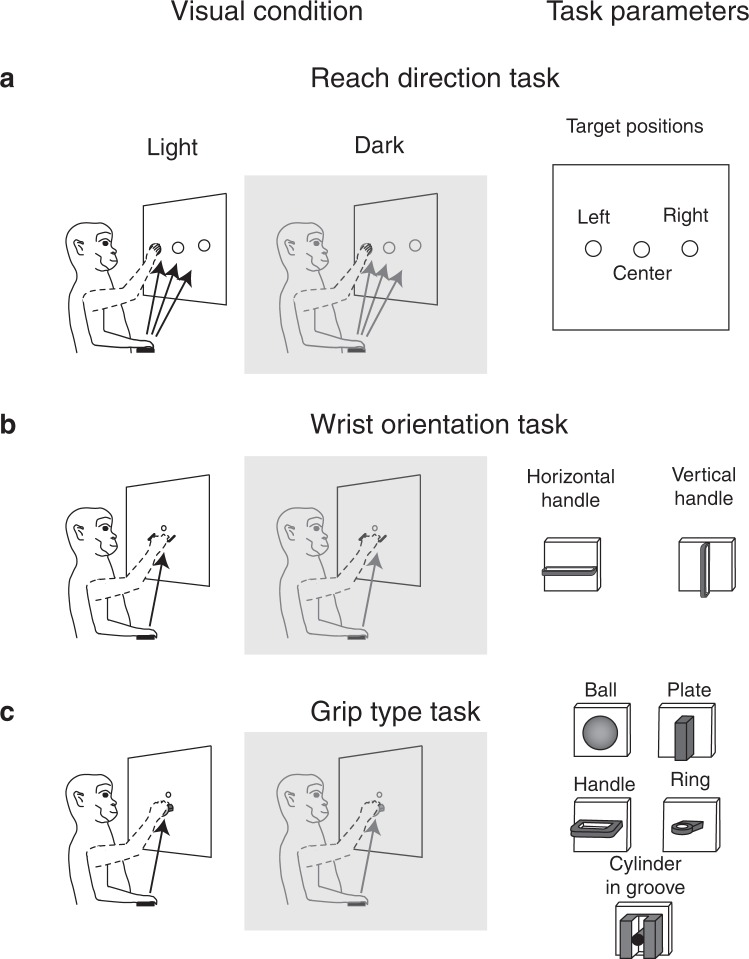
The three tasks with different types of arm/hand actions used to test V6A neural population (**a**) Reach direction task. Left, Reaching movements were performed in the light and in the dark (gray shade) background, from a home button (black rectangle) toward one of three targets located on a panel in front of the animal. Right, frontal view of the three target positions on the frontoparallel panel. (**b**) Wrist orientation task. Left, grasping movements were performed in the light and in the dark (gray shade) background, from a home button (black rectangle) toward a handle located in the same spatial position in front of the animal. Right, frontal view of the handle that could be horizontally (left) and vertically (right) oriented. (**c**) Grip type task. Left, grasping movements were performed in the light and in the dark (gray shaded) background, from a home button (black rectangle) toward five different objects. Right, frontal view of the ball, plate, handle, ring and cylinder in groove.

**Figure 2:**
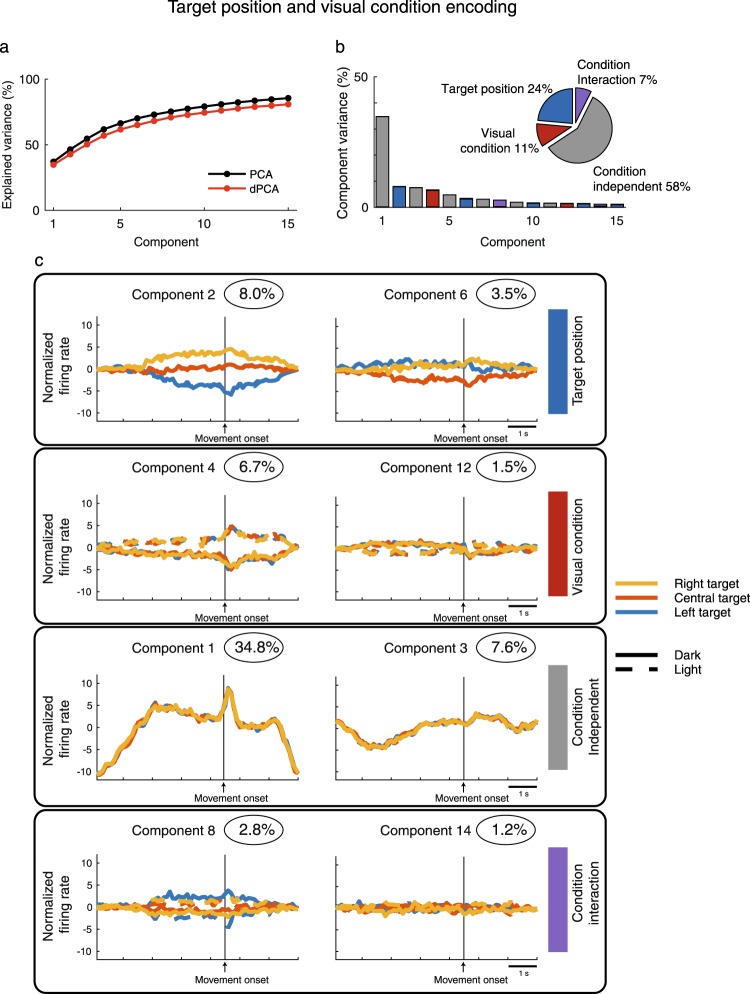
Demixed principal component of reach direction task. (**a**) Cumulative signal variance explained by PCA (black) and dPCA (red). dPCA explains almost the same amount of variance as standard PCA. (**b**) Variance of the individual demixed principal components. Each bar shows the proportion of total variance. Pie chart shows how the total signal variance is split between parameters (visual condition and target position). (**c**) Time course of the projections of single components aligned on movement onset. From top to bottom, each panel of projections of components is relative to: target position, visual condition, condition-independent and condition interaction.

A population of 149 neurons were recorded from area V6A in two monkeys while the animals performed a *reach direction task* toward three different positions in the light and in the dark^12^ (Fig. 1a). For each of these conditions (3 target positions and 2 visual conditions) and for each trial, we extracted the neural activity from −4500 ms before the reaching onset to 2500 ms after it, that is for all the duration of the trial. To get a population-level picture of the effect of visual background and target location on neural activity, we used a demixed principal component analysis (dPCA) that not only reduced the dimensions of the data, but also demixed the dependencies of the population activity on the task parameters. Figure 2a shows that the amount of variance explained by dPCA was consistent to that extracted by the classical PCA. Figure 2b shows that the amount of population variance could be decomposed in four distinct categories: the first category was the target position, that captured 24% of the total variance of neural population; the second was the visual condition, with 11% of the total variance captured; the third, the condition-independent, with 58% of the total variance captured; and the forth, dependent on the interaction of the two variables, that captured 7% of the total variance. The time course of the first two components representing each category described above is shown in Fig. 2c. Each subplot in Fig. 2c shows the projections of the peri-stimulus time histograms (PSTH) of all neurons onto the most prominent axes; they are represented as 6 lines corresponding to the 6 conditions analyzed (3 target positions × 2 visual conditions). Because the projections of the component relative to target position (component 2, Fig. 2c, first row) were clearly separated, V6A cells were likely to show spatial tuning that persisted during the planning phase of the movement and its execution and holding of the reaching target (component 2). Notice that the separation between the two components was enhanced during the movement execution. The segregation of projections of component 4 (visual condition, Fig. 2c, second row) indicates that V6A cells were able to encode the visual condition for the entire duration of the trial especially during movement execution (see the changing of projection slopes aligned with the onset of movement). Component 1 represents the projections not dependent from the two parameters considered, but showed a notable correlation with the movement execution (Fig. 2c, third row). Overall, Fig. 2 shows that, during the reaching, the amount of population variance encoding the spatial parameters driving the arm to the intended spatial location of target was higher than the amount of population variance encoding the visual information. This suggests that the population was mainly involved in the target position encoding rather than in the visual condition, and this persisted from the planning phase of the movement to the end of trial.

**Figure 3:**
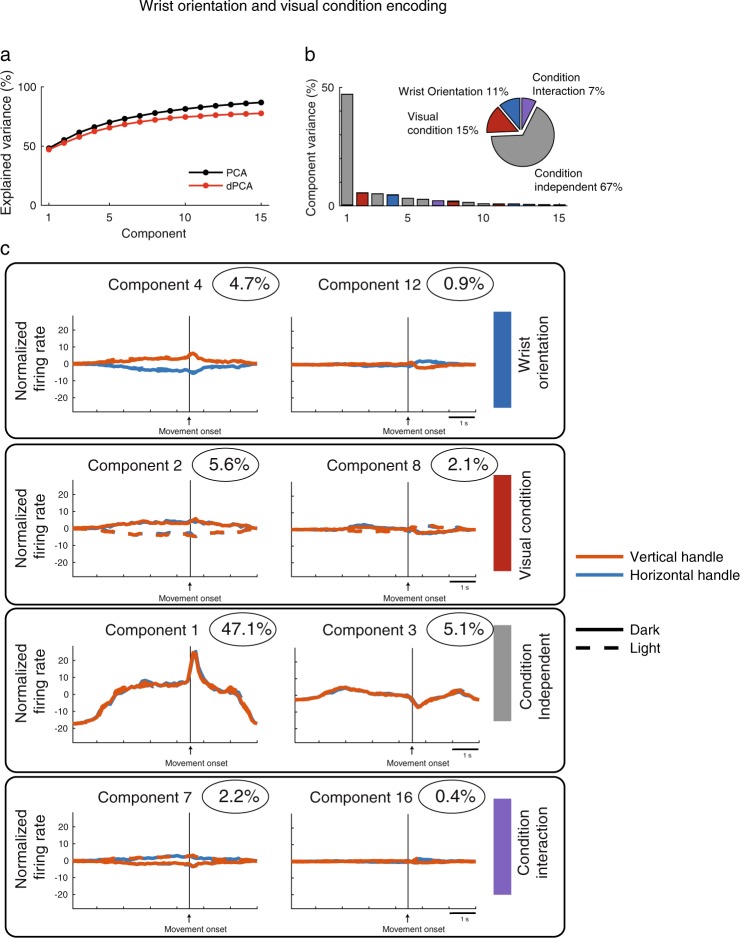
Demixed principal component of wrist orientation task. All conventions are as in Fig. 2.

To analyze the contribution of visual condition and wrist orientation on the activity of V6A population, we analyzed the activity of 262 V6A neurons in two monkeys during the execution of the *wrist orientation task* in light and in dark. The *wrist orientation task* consisted in reach-to-grasp movements towards a handle, which was located in a constant spatial position, that could be horizontally or vertically oriented in order to require different wrist orientations to match the object during grasping. Each neuron was tested with two handle orientations and in two visual conditions (Fig. 1b). As for the reaching task, the consistence between the variance captured by traditional PCA and dPCA analysis was high (Fig. 3a). The portions of total variance captured by the neural population are shown in Fig. 3b: the 67% of the total variance captured was not correlated with the observed parameters, the visual condition and wrist orientation captured the 15% and 11% of the total variance, respectively, and the 7% of the total variance represented the interaction between conditions. Figure 3c shows that the projections of components relative to wrist orientation (Component 4, first row) were well separated from ~1500 ms after the beginning of the trial until ~500 ms after the movement onset, and then tended to converge after grasping execution. Similarly to reaching data, visual information components (Component 2, second row) were well separated for the entire duration of the task, suggesting a powerful ability of neural population in sustaining the discrimination between the two visual conditions across time. The condition independent projections (Component 1, third row) was correlated to the movement execution, similarly to what occurred in reaching, whereas the projections of condition interaction (Component 7, bottom row) followed the same trend as the projections representing the visual condition. In the wrist orientation task, the proportion of variance captured by wrist orientation and visual condition (11% and 15%, respectively) was smaller than the variance captured by the condition-independent component (67%), suggesting that only a small proportion of neural population was encoding the two task parameters.

**Figure 4:**
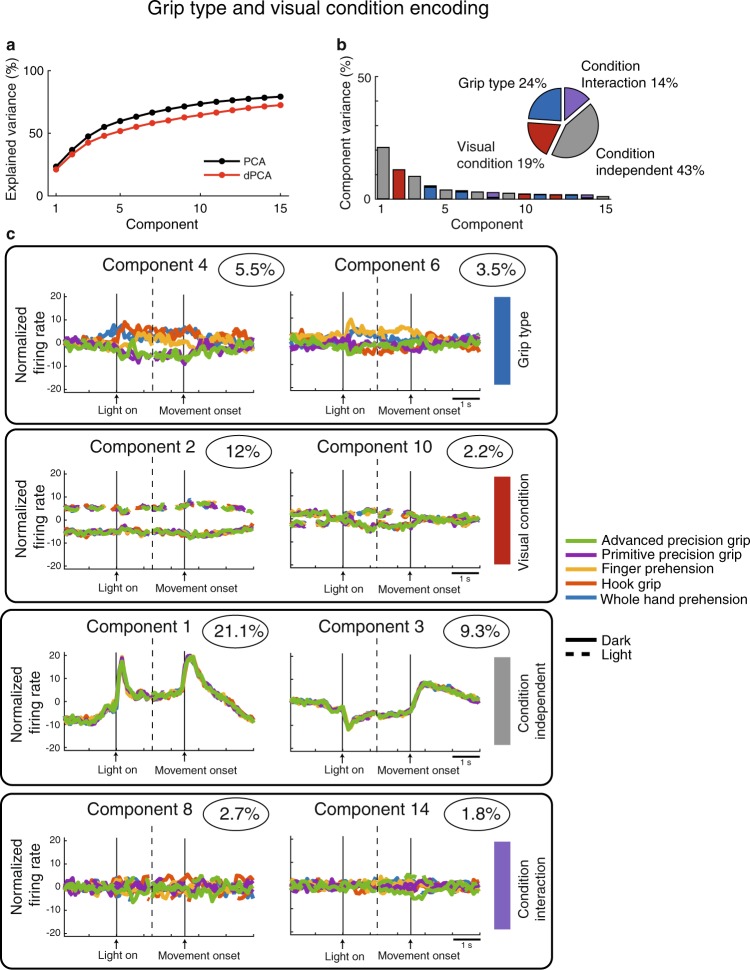
Demixed principal component of grip type task. The time course of the projections of single components in c) are aligned on the object illumination and on movement onset (2 black arrowheads). Dashed lines indicate the interruption of the component projection to obtain the double alignement. Other conventions are as in Fig. 2.

A population of 317 neurons were recorded from V6A in the *grip type task* in light and dark conditions. Two monkeys were required to grasp objects with five different shapes, thus requiring five different grips. The objects were a “ball” grasped with “whole-hand prehension”, a “handle” grasped with “finger prehension”, a “ring” grasped with the “hook grip”, “plate”, grasped with the “primitive precision grip” and a “cylinder-in-groove” grasped with the “advanced precision grip” (see^14^ for details). Notice that these five objects evoked different grips but in the same spatial position and with the same wrist orientation (Fig. 1c). Once again, the five objects were grasped either in dark or in light. We used dPCA analysis to evaluate which proportion of V6A population contributed to encode the grip types and the visual information. Similarly to what found for *reach direction* and *wrist orientation tasks* (Figs 2a and 3a), the amount of variance explained by dPCA was very similar to that extracted by classical PCA (Fig. 4a). The condition independent components represented the largest proportion of variance captured by our population (43%, Fig. 4b). Visual information and grip type captured 19% and 24% of the total variance, respectively, and the condition interaction was represented by 14% of the total variance (see Fig. 4b). The projections of grip type component (Component 4, Fig. 4c, first row) show that the population discriminates two groups of prehension types in an interval that ranges from the illumination of the object until the end of movement execution (~500 ms after movement onset). The first group is represented by the whole hand prehension, the hook grip and the finger prehension, the second group by primitive and advanced precision grip. The two visual conditions were well discriminated by the population for the entire duration of the trial (Component 2, Fig. 4c, second row). The condition-independent component (Component 1, Fig. 4c, third row) was strongly correlated with the object illumination and the execution of movement. The time course of the condition interaction component (Component 8, Fig. 4c, bottom row) did not show significant tuning. In this task, the proportion of population involved in the encoding of grip type is equal to that encoding target position in the *reach direction task* (24% in both tasks). The difference is evident in the observation of the time course of the components. In the *reach direction task*, the population discriminated the three individual target positions as the three lines are well separated during the trial execution (Fig. 2c, first row, Component 2). In the *grip type task*, the time course of component 4 (Fig. 4c, first row, Component 4) is divided in two clusters evoking, on one side, more skilled prehensions and, on the other, coarser ones.

The component projections displayed in the right column of each panel in Figs 2c, 3c and 4c resemble derivatives of the component projections discussed above that are due to slight variations in the timing of responses across neurons (see Appendix B of^22^).

**Figure 5:**
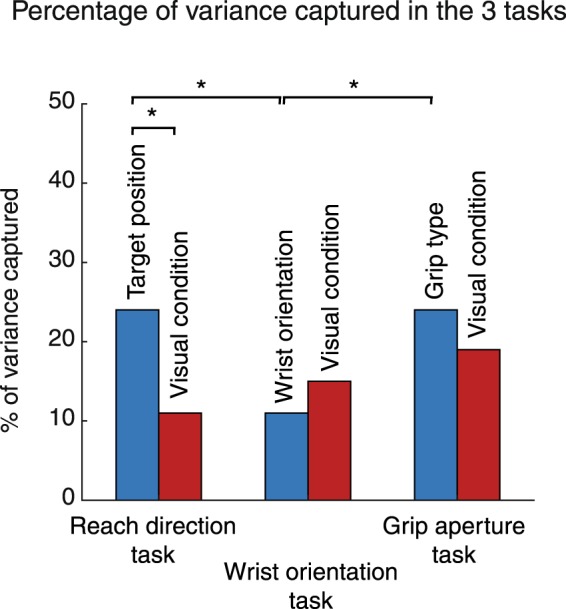
Distribution of variance captured in the three tasks. Blue columns show the percentages of variance captured by target position, wrist orientation and grip type parameters of the three tasks. Red columns show the percentages of variance captured by visual condition in reach direction, wrist orientation and grip type tasks, respectively. Asterisks indicate significant differences (Chi-squared, P < 0.05).

Figure 5 summarizes the comparison of percentages of variance captured in the three tasks we analysed. The amount of variance relative to the visual conditions was not statistically different from one task to the other (11%, *reach direction task*, 15%, *wrist orientation task*; 19%, *grip type task*; chi-squared, P > 0.05). The distribution of variance corresponding to target position was significantly higher than those of wrist orientation (24%, *reach direction task vs* 11%, *wrist orientation task*, chi-squared, P < 0.05) and not significantly different from grip type (24%, *reach direction task* vs 24% *grip type task*, chi-squared, P > 0.05). The grip type parameter was significantly higher than the wrist orientation one (24%, *grip type task* vs 11% *wrist orientation task*, chi-squared, P < 0.05). The distribution of the task variables within each task displayed significant differences only in the *reach direction task* (11%, visual condition vs 24%, target position, chi-squared, P < 0.05).

To check whether the comparison among the three tasks was reliable, we performed the dPCA analysis with the same three neural populations but with a symmetrical 2 × 2 experimental design (2 target positions × 2 visual conditions; 2 wrist orientations × 2 visual conditions; 2 grip types × 2 visual conditions). We found that the neural populations tested in the three tasks clearly discriminated the task specific parameters (target position, wrist orientation and grip type, respectively) and the two visual conditions (Fig. 1S). Interestingly, the proportion of total variance captured by the variables considered was consistent with the proportion of variance captured using the total set of task variables (see Fig. 2S).

### Minimun population size for reliable demixing

**Figure 6:**
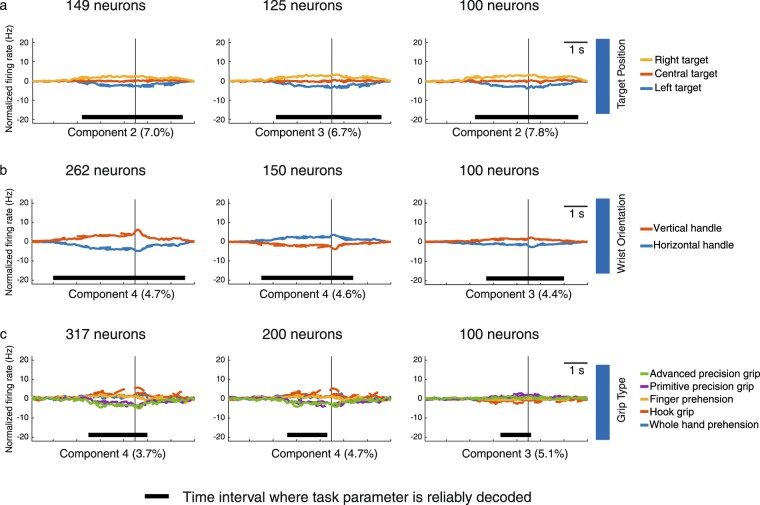
Minimum population size for reliable demixing. (**a**) Time course of the projections of single components aligned on movement onset relative to target position extracted, from left to right, from the total population, and from subpopulations of 125 and 100 neurons tested in the *reach direction task*. (**b**) Time course of the projections of single components aligned on movement onset relative to wrist orientation extracted, from left to right, from the total population, and from subpopulations of 150 and 100 neurons tested in *wrist orientation task* (**c**) Time course of the projections of single components aligned on movement onset relative to grip type extracted, from left to right, from the total population, and from subpopulations of 200 and 100 neurons tested in *grip type task*. Black horizontal thick lines indicate the time intervals where task parameters are reliable decoded.

To find the minimum number of V6A cells needed to obtain reliable estimates of the demixed components, for each of the three tasks, we extracted 100 neurons from the entire population, that represent the minimum number recommended by Kobak and co-authors^22^, and an amount of neurons specific for each task (125 neurons in *reach direction task*; 150 neurons in *wrist orientation task*; 200 neurons in *grip type task*, respectively). Then, we evaluated the reliable demixing on the task parameters specific for each task by calculating the time intervals where the components were statistically tuned or decoded through the use of a linear classifier and a cross-validation procedure as described in Kobak *et al*.^22^. We used the linear classifier to measure time-dependent classification accuracy and a shuffling procedure to assess whether it was significantly above the chance level (see^22^). From the time intervals of significant tuning (marked with horizontal black lines in Fig. 6), we found that in *reach direction task* the neural population was significantly tuned without interruptions from the planning phase to the movement execution and target holding (from ~2400 ms before the movement onset to ~2000 ms after it). This occurred both when testing the entire population as well as the 125 and 100 neuron supopulations (Fig. 6a). In *wrist direction task*, when testing the entire population, the wrist orientation tuning was significantly persistent from ~3500 ms before the movement onset to ~2200 ms after it (Fig. 6b, first diagram). For the subpopulations with 150 and 100 neurons the result was similar, although with slightly shorter periods of statistical difference (Fig. 6b, second and third diagram). In the *grip type task*, the best tuning was significantly extracted from the entire population from ~2000 ms before the movement onset to ~500 ms after it, as shown in Fig. 6c (first diagram). The other 2 neural subpopulations (200 and 100 neurons) presented evident shorter tuning (Fig. 6c, second and third diagram). Overall, these results suggest that the minimum population size for a reliable demixing is 100 neurons in the *reach direction* and *wrist orientation tasks*. In contrast, in the *grip type task* all neurons are necessary to obtain an optimal demixing, probably because of the higher number of task parameters.

### Time-course of parameter discrimination of V6A population in the three tasks

**Figure 7:**
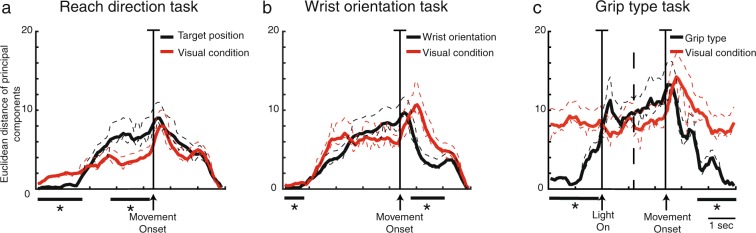
Time course of the discrimination ability of V6A neural population. (**a**) *Reach direction task*. The time course of the discrimination ability is calculated as euclidean distance of the principal components and results in two curves. The two curves correspond to discrimination ability of the two target positions (black curve) and the two visual conditions (red curves). Dotted curves represent lower and upper confidence intervals that include 95% of resampled values. (**b**) *Wrist orientation task*. The two curves correspond to discrimination ability of the wrist orientation (black curve) and the two visual conditions (red curve). (**c**) *Grip type task*. The two curves correspond to discrimination ability of the grip types (black curve) and the two visual conditions (red curve). The curves related to reach direction and wrist orientation tasks are aligned at the onset of movement (arrow). The curves related to *grip type task* are aligned on the object illumination and on movement onset (2 arrows). Asterisks indicate intervals of significant separation calculated by a randomization test (P < 0.05, 100 iterations).

Figures 2c, 3c and 4c show that the components related to visual information and to the other task variables (spatial position, wrist orientation, grip type) were well separated along the entire duration of the analysed time interval. To better evaluate the relative strength of the discrimination between visual condition and each of the other task variables along the time-course of the trial, we computed the euclidean distance of the time between the projections of the component representing visual condition and those representing each of the other task variables (Fig. 7). In order to make the three tasks comparable, we used a constant design of visual condition and task parameters (2 visual conditions × 2 task parameters). For *reach direction task* we used the left and right targets whereas for *grip type task* we used the whole hand prehension and the advanced precision grip.

In the *reach direction task*, we calculated the mean curve between the projections of the component corresponding to the left target in light and that corresponding to the left target in dark, and we did the same also for the right target. Then, we extracted the euclidean distance between these two mean curves to evaluate how the population discriminates between left and right targets across the task. Similarly, we calculated the mean curves for the projections of the components corresponding to dark and light conditions and we extracted the euclidean distance between the two curves. The latter curve displays how visual condition was discriminated by neural population along the task. The course of the two euclidean distances is reported in Fig. 7a. To determine when the two curves were statistically separated, we resampled each value corresponding to each bin in order to obtain lower and upper confidence intervals (CI) that included 95% of values. We considered a statistical separation of the curves be significant when the corresponding CIs did not cross each other and when this separation was maintained for at least 10 consecutive bins. In Fig. 7, the significant intervals are maked with black lines and asterisks. V6A population discriminated the two target positions and the two visual conditions with similar time evolution after the onset of movement (Randomization test, P > 0.05), clearly increasing the discriminative power ability at the movement onset (alignment line). During the planning phase, the discrimination of target position was significantly higher than that of visual condition. The visual condition was significantly discriminated only at the beginning of trial. This result suggests that the population processed similarly the spatial and visual information after the movement onset and for the entire remaining duration of the trial.

In Fig. 7b, the same analysis is shown for the V6A population tested with the *wrist orientation task*. We calculated the euclidean distance between the mean projections of components showed in Fig. 3c that represent the wrist orientation and the visual condition, respectively. The discrimination of the visual condition was significantly higher than the wrist orientation one in the first 800 ms of the trial and 500 ms after the movement onset (Randomization test, P < 0.05). During the planning phase, the discrimination powers of the visual condition and wrist orientation were similar, with a final take over (from 3700 ms before the movement onset to 500 ms after it) of the visual condition encoding. This occurs after the completion of the grasp, during hand holding.

This trend was different for the population tested in the *grip type task*. We calculated the mean euclidean distances of components representing the grip type and visual information, respectively. In Fig. 7c, the euclidean distance analysis shows that the population discriminated similarly the visual condition and the grip type during the time period between object illumination and ~1000 ms after the movement onset. The discrimination of visual condition was statistically higher during the first ~2100 ms of the trial and from ~1000 ms after the movement onset to the end of trial (Randomization test, P < 0.05). Thus, the two grip types and the visual conditions were encoded similarly during the task-relevant phases of trial (from the vision of object to be grasped to the grasping action). However, similarly to what occurred in the wrist orientation task, the visual condition was more significantly encoded after grasp execution.

## Discussion

In this work, we aimed at investigating how V6A encodes visual information and different reach-to-grasp parameters, as well as their interaction in a prehension task, by a dimensionality reduction technique. By this approach, we have obtained a picture of the neural population that highlights novel aspects of the encoding process not evident through the single-cell analysis. To obtain this picture, we used three different V6A populations recorded in three different tasks according to the same used in the previous studies^12–14^. Although the ideal approach would be to have a unique neural population, a recent study comparing the neural population features of area AIP and F5 by dPCA using two distinct populations^23^ supports the idea that it is reliable to extract a unique neural feature from different groups of neurons. In the present study, to go beyond the limitations of having different neural populations, we performed a dPCA analysis considering symmetrical number of variables in all tasks (see Supplementary Information). In this way, we added robustness to the overall results reported in the present work. In our case, the results are in agreement with those of previous studies^12–14^ suggesting the ability of the dPCA tecnique in extracting the most relevant population features. It is worthwhile to notice that we used the entire recorded populations without any preselection. In this way, we were able to reproduce the same sampling criteria that would fit with prosthetic applications, where task-related and unrelated neurons are used^24–26^.

The three tasks we used encompassed all the components of prehensile actions: transporting the hand towards different spatial positions, adjusting wrist orientation to orient the hand to match the object orientation, adapting the hand to accomplish the more suitable grip to grasp objects of different shapes. In addition, the three tasks were accomplished with different availability of visual information: in the light, with full vision of the target of action during both motor preparation and execution, as well as of the hand approaching the object and interacting with it, and in the dark, that is without any visual feedback.

Previous reports demonstrated that, in cognitive tasks, the task-relevant aspects and their combination are distributed across multiple neurons in an explicit format^20,27^, and that this format is easily accessible to linear classifiers, such as simple neuron models^28,29^. Based on this evidence, we applied a dPCA method to verify how relevant parameters of the tasks are distributed across the V6A neural population and how they are temporally encoded across the progression of the task. This approach allowed us to observe how the entire neural population dealt with the task parameters without the bias due to prior selection of neural activity.

While we found that the amount of variance due to visual information was similar in the three tasks we tested, an increasing trend of variance was observed according to wrist orientation (11%), grip type (24%), and reaching direction (24%). The significant differences found in the incidence of these three task parameters could reflect the difficulty and computations required by the three types of actions. Note that the *wrist orientation task* required only a change in wrist orientation, according to the orientation of the handle to be grasped, and not a spatial computation of object position, the object being always in the same spatial location; nor a change in grip type, the object shape being constant, so requiring the same type of grip. This could explain the low influence of this parameter on the variance. Grasping different objects (*grip type task*), instead, called for different finger ‘pointing’ combinations, thus compelling higher complexity and precision that would influence the population variance in a more influential manner. In the *reach direction task*, the amount of variance captured by target position was the same as in the grip type task, and higher than in the *wrist orientation* task. This difference can be explained by the requirement of a large computational load as the hand is directed in the space to accurately point a small visual target, or to accurately preshape the fingers around an object, though this was not reported in previous studies conducted at single cell level^12–14^. Another explanation could be related to the different training level of the monkeys performing the *wrist orientation task* with respect to the *reach direction* and *grip type tasks*. Another aspect that could potentially explain the trend depicted, could be the unbalanced number of conditions tested in the three tasks (2 for wrist orientation, 3 for reach direction and 5 for grip type), so in the Supplementary Information we performed dimensionality reduction considering 2 conditions for every task: the relative weight of the different task parameters (target position, wrist orientation and grip type) remained consistent also when the dPCA was performed considering two variables in all tasks (see Supplementary Information). On one side, this result demonstrated that the encoding of task-specific parameters depended on task requirements and not on the number of conditions considered. On the other side, the reduction of the dimensions of neural population shows to be a reliable tool to compare and extract the relevant features of the neural activity during different tasks.

Recent human studies have suggested that the regions of the dorso-medial circuit hosting V6A are involved in the encoding of both grip type and directional components of arm movement^30–32^. Recently, Hatsopoulos’ lab^33^ demonstrated that PMd neurons are modulated by proximal and distal components of prehension. They showed that PMd neurons encode the kinematics of both reaching and grasping synergies, in agreement with previous studies showing that the premotor cortex is involved in the control of the entire prehension^34,35^. Area V6A is strictly connected with area PMd^36^. The spatial coordinates about hand and target position could be sent by V6A to PMd, to generate the motor plans. Copies of the resulting motor plans could be sent back, as corollary discharge, to V6A, to compare the desired position of moving limb with the actual one. It has been supposed that this dorsomedial parieto-frontal circuit including V6A and PMd shares functional similarities in order to control the prehension on-line^3,4,19^. Indeed, the same three factors investigated in V6A in the present study (target position, wrist orientation and grip type), are encoded also in PMd^34,35^, suggesting a strict functional interaction within the dorso-medial fronto-parietal pathway^37^.

The demixing of grip type confirmed that V6A population encoded the different grips^1^, but also showed that the specific motor responses depended on the use of index finger, as the grip types were clustered in two groups (Fig. 4c, see component 4). These results are in accordance with those found in Fattori *et al*.^10^, where the cluster analysis showed significant combination of grip types characterized by the use of the index finger. In the present work, it is clear that the encoding of grip types sharing similar features started before the onset of movement, specifically from the object illumination^38^, and continued until the hand approached the object. These findings suggest that V6A encodes the common features of hand grips required to grasp different objects.

The present work also investigated the time course of the components representing the visual conditions and the task-specific parameters. V6A cell population was able to discriminate between the considered variables. In previous studies^12–14^, we demonstrated that V6A neurons were selective for both the visual condition and task parameters (target position, wrist orientation, grip type), but a direct comparison of discrimination ability of neural population along trial progression and among the three different tasks has never been done. Although the time courses of the different discrimination abilities were clearly visible along trial progression in the three different tasks in Figs 2c, 3c, 4c, the computation of the euclidean distance between the corresponding components (Fig. 7) allowed us to highlight the major differences according to the task type. In the *reach direction task*, we found that the time course of discrimination ability between the two visual conditions was completely overlapped only after the movement onset (Fig. 7a). The spatial position of the target was better discriminated during the planning phase of reaching. A possible explanation for these results is related to the importance of visual information in reach monitoring: before and after the movement, visual information is less relevant than spatial information as it is necessary to accurately direct the hand to the target^39^.

In the *wrist orientation task*, the time course of parameter discrimination between the two visual conditions and the two wrist orientations followed the same pattern during the planning phase of arm movement, but after the movement onset the visual conditions became more discriminated with respect to the wrist orientations (Fig. 7b). These results demonstrate that visual condition and wrist orientation have different weights in the information processings according to the different phases of the task. The visual condition was strongly discriminated during and after the execution of movement, likely to monitor potential adjustments of wrist posture before and perhaps just after having performed the grasp. This parallels psychophysical studies demonstrating that the visual feedback is used both to guide the hand and the fingers toward the final grasping position^40^ and to determine the relative distance beetween hand and object^41^. Moreover, it was demonstrated that using a task in which participants were asked to initiate reaches toward multiple potential targets that varied both in location and orientation, the initial movement trajectory took into account not only the locations of targets, but also their orientations^42^. As the initial part of movement trajectory reflects the movement plan, the overlap between the discrimination pattern of wrist orientation and visual condition found during the planning phase of the task in our study suggests that a similar encoding of both kinds of information before the movement occurrs at both behavioral and neural level.

In the *grip type task*, the time courses of visual information and grip type parameter followed the same evolution in an interval that covers the most relevant phases of task: from the acquisition of visual information of the object features (object illumination) to the holding of the object. Then, the visual condition remained more discriminated for the remaining part of the trial. Again, as for wrist orientation, feedback monitoring of object holding in the hand after grasp completion seems to be a typical feature of V6A population. This alternation of discrimination ability of task variables during task progression agrees with the view that each phase required different weight of information processing (visual information and proprioceptive/tactile combination in the planning and motor phases of the task).

The monkey medial PPC has been recently considered as a site that encodes intentions for reaches, and neural signals from this cortical region have been proposed to be applied in neuroprosthetics research^25,26^. Several areas of the PPC, not only area V6A, have been reported so far to be involved in the control of prehension. However, to our knowledge, only V6A has been tested in reaching and grasping actions where encoding of target position/arm movement direction, wrist orientation, and grip type are investigated. Other areas of the medial PPC have been reported to encode some parameters of reaching, as the target position/arm movement direction (area PRR -parietal reach region and area PEc-caudal superior parietal^43–48^), but nothing has been reported so far about a putative involvement of these areas in encoding specific parameters related to grasping, as wrist orientation or grip type. In the more lateral PPC area AIP (anterior intraparietal area), neurons encode wrist orientation and grip type^49–51^, but the possible encoding of target position/arm movement direction has not been fully investigated. Although, at present, a comparative study on the relative weight of the parameters of prehension in the different areas of the medial PPC is still lacking, it seems that PRR and PEc are involed in encoding spatial parameters for reaching and AIP in the encoding of object-oriented pragmatic actions, with V6A well suited to coordinate arm movements and grasping^19^. Decoding approaches showed that V6A can be a good source for decoding directional signals for reaching^24^ and recently neural signals from V6A have been shown to be suitable for decoding hand shaping useful to implement brain machine interfaces to optimally guide prehensile actions^52^. The dimension reduction performed in the present study represents a general frame of the functional properties of the medial posterior parietal cortex that may be essential for improving computational efficiency in handling massive amounts of neural data for application in neuroprosthetics^53,54^.

## Materials and Methods

### General procedures

Experiments were carried out in 4 *Macaca fascicularis* (males, weight 3–7 Kg). The study was performed in accordance with the guidelines of EU Directives (86/609/EEC; 2010/63/EU) and Italian national laws (D.L. 116-92, D.L. 26-2014) on the protection of animals used for scientific purposes. Protocols were approved by the Animal-Welfare Body of the University of Bologna. During training and recording sessions, particular attention was paid to any behavioral and clinical sign of pain or distress.

All the surgical and postoperative procedures were previosly reported^55^. Extracellular recording techniques, reconstruction of microelectrode penetrations, the criteria used to recognize V6A, and the experimental setup used here were as described in previous reports^56–59^.

### Behavioral Tasks

#### Reach direction task

Two monkeys performed instructed-delay reaching movements directed outward from the body to visual targets represented by left, central and right target positions. The task was executed in two conditions: in the dark, when the animal could only see the target, and in the light, when the target, the moving arm, and the environment were visible. As shown in Fig. 1a, reaching movements started from a button located outside the animal’s field of view. Reaching movements transported the hand from the home button to targets positioned in three different spatial positions at eye level (left, center and right target position, Fig. 1a-right) on a frontoparallel panel. The details of trial sequence are reported in Bosco *et al*.^12^.

#### Wrist orientation task

We tested V6A cells while two monkeys performed instructed-delay reach-to-grasp movements towards a handle located in a fixed spatial position on a frontal panel, while the gaze was stable on it^9,13^. As shown in Fig. 1b (left), the *wrist orientation task* was performed in the light and in the dark as in the *reach direction task*. Figure 1b-right shows the two different orientations assumed by the handle (horizontal and vertical), so the monkey approached and grasped it with different wrist orientations: hand pronated and half pronated, respectively. In both cases, the monkeys grasped the handle with the same grip: finger prehension. The time sequence of wrist orientation task is reported in Beveglieri *et al*.^13^.

#### Grip type task

Two monkeys performed instructed-delay reach-to-grasp movements towards objects with five different shapes. The object shapes are reported in Fig. 1c (right). The animals performed the reach-to-grasp task in light and dark conditions as shown in Fig. 1c (left). The task began when the monkey pressed the home button. All details related to different grip types and task sequence are reported in Breveglieri *et al*.^14^.

We used 2 visual conditions and 3 target positions in the reach direction task, 2 visual conditions and 2 wrist orientations in the wrist orientation task and 2 visual conditions and 5 grip types in the grip type task, thus the same conditions as those of previous studies^12–14^.

### Data analysis

Custom scripts in Matlab were used to perform all the analyses (Matworks, Natick, MA, USA, RRID:SCR_001622). We quantified the neuronal activity in each trial during the three tasks in dark or in light. For each condition (3 target position/2 handle orientations/5 object shapes and 2 visual conditions) and for each trial, we extracted the neural activity from −4500 ms before the onset of reaching movement to 2500 ms after it, in bins of 40 ms. This time interval includes all the duration of the trial. In the *reach direction* and *wrist orientation tasks*, the least trial number was 7 and the most was 15. In the *grip type task* the minimum number of trials was 7 and the maximum was 10.

For each of the three tasks we tested, we performed a dPCA analysis^22^. This analysis allowed us to reduce the dimensionality of the data as the standard PCA but demixing all task parameters controlled or monitored by the experimenter (i.e. sensory and motor variables). In PCA, the original data (firing rates of the neurons) are linearly transformed into several latent variables, called principal components. Each principal component is a linear combination, or weighted average, of the actual neurons. We interpret these principal components as linear “read-outs” from the full population (“decoder” D). This transformation can be viewed as a compression step, as the number of informative latent variables is usually much smaller than the original dimensionality. The resulting principal components can then be de-compressed with another linear mapping (“encoder” F), approximately reconstructing the original data. Geometrically, when applied to a cloud of data points, PCA constructs a set of directions (principal axes) that consecutively maximize the variance of the projections; these axes form both the decoder and the encoder.

More precisely, given a data matrix X, in which each row contains the firing rates of one neuron for all task conditons, PCA finds decoder and encoder that minimize the loss function:

$${L}_{PCA}={\Vert X-FDX\Vert }^{2}$$

under the constraint that the principal axes are normalized and orthogonal, and therefore D = F^T ^^60^. However, applying PCA to a neural dataset generally results in principal components exhibiting mixed selectivity. dPCA does not only compress the data optimally, but also demixes the selectivities. As in PCA, compression is achieved with a linear mapping D and decompression with a linear mapping F. Unlike PCA, the decoding axes are not constrained to be orthogonal to each other and may have to be non-orthogonal, in order to comply with demixing. dPCA first splits the data matrix into a stimulus-varying part X_s_ and a time varying part X_t_, so that X ≈ X_t_ + X_s_, and then finds separate decoder and encoder matrices for stimulus and time by minimizing the loss function:

$${L}_{dPCA}=||{X}_{s}-{F}_{s}{D}_{s}X|{|}^{2}+||{X}_{t}-{F}_{t}{D}_{t}X|{|}^{2}$$

The free code is available at: http://github.com/machenslab/dPCA^22^.

To compare the distributions of percentages of variance captured by the task variables within each task and across tasks, we used the chi-squared test (P < 0.05).

To assess the minum number of neuron population in each task that allows a reliable demixing by dPCA analysis, in each task, we randomly extracted two different subpopulations with respect to the original population of neurons. In all tasks, we randomly extracted 100 neurons, representing the minimum number recommended by Kobak and co-authors^22^ and an intermediate amount of neurons that was specific to each task (125 neurons in *reach direction task*; 150 neurons in *wrist orientation task*; 200 neurons in *grip type task*, respectively). We used the decoding axis of each dPCA in the three task parameters marginalizations as a linear classifier to decode the target position, the wrist orientation and the grip type, respectively. We used 100 iterations of stratified monte Carlo leave-group-out cross-validation, where on each iteration we held out one trial for each neuron in each condition as a set of SQ test “pseudo-trials” X_test_ and averaged over remaining trials to form a training set X_train_. After running dPCA on X_train_, we used decoding axes of the first task parameter dPCA as a linear classifier to decode target position, wrist orientation and grip type, respectively^22^. We repeated this procedure three times for each task that correspond to all population sets tested.

To compare the discriminative ability of the three V6A populations, we calculated the euclidean distance between the averaged components representing the visual information and each of the remaining task variables, that are the target position, the wrist orientation and the grip type, respectively. In particular, for *reach direction task*, we calculated the euclidean distance between the components representing the left and right targets averaged across light and dark and the euclidean distance between the components representing the light and dark averaged across left and right position, respectively. We computed the distances along the entire duration of the task interval. In this way, we obtained two curves, one representing the discrimination ability between the two visual conditions and the other the discrimination ability between the two targets. For the *wrist orientation and grip type tasks*, we followed the same procedures considering the wrist orientation and the grip type components, respectively. To determine when the two curves were statistically separated, we resampled each value corresponding to each bin by a randomization test (100 iterations, P < 0.05) to obtain lower and upper CIs that included 95% of values. We considered a statistical separation of the curves when the corresponding confidence intervals did not cross each other and this separation was maintained at least for 10 consecutive bins.

## Supplementary information

Supplementary Information

## Data Availability

The data that support the findings of this study are available from the corresponding author upon reasonable request.
