# Supplementary material for: Reduced neural representation of arm/hand actions in the medial posterior parietal cortex
Source: Sci Rep. 2019 Jan 30;9:936. doi: 10.1038/s41598-018-37302-2 (PMC6353970; doi:10.1038/s41598-018-37302-2)
Supplement: Supplementary file 1 — Supplementary Information [file 41598_2018_37302_MOESM1_ESM.pdf]

Reduced neural representation of arm/hand actions in the medial posterior parietal cortex.

Bosco A.[1], Breveglieri R.[1], Filippini M.[1], Galletti C.[1], Fattori P.[1*]

[1]Department of Biomedical and Neuromotor Sciences, University of Bologna, Italy

[*]**Corresponding author**

E-mail: patrizia.fattori@unibo.it.

Running title

Neural representation of arm/hand movements in the medial PPC.

Author contribution statement

P.F. and C.G. designed the research; A.B., R.B. and M.F. recorded the data; A.B. analysed the data; A.B., P.F., R.B. and C.G. wrote the paper with inputs from all authors. All authors contributed to this work, read the manuscript, discussed the results, and all agree to the contents of the manuscript.

Keywords

Motor response, visual feedback, reaching, grasping, neural activity, principal component analysis.

Data availability

The data that support the findings of this study are available from the corresponding author upon reasonable request.

Acknowledgment: This work was supported by Firb 2013 N. RBFR132BKP (MIUR) and by Platypus (EU H2020-MSCA-RISE-2016 734227). We thank Michela Gamberini and Lauretta Passarelli for the anatomical reconstructions.

Competing interests

The authors declare no competing interests.

SUPPLEMENTARY INFORMATION

MATERIALS AND METHODS

Behavioral tasks and data analysis

The tasks performed were the same described in the main manuscript, but we performed the dPCA analysis using the same number of conditions for the three tasks considered: 2 visual conditions and 2 target positions (left and right targets) in the *reach direction task*, 2 visual conditions and 2 wrist orientations (horizontal and vertical orientations) in the *wrist orientation task* and 2 visual conditions and 2 grip types (whole hand prehension and precision grip) in the *grip type task*.

RESULTS

The dPCA analysis performed on the population recorded in the *reach direction task* shows that the total amount of variance captured is distributed as following: 22% was captured by the target position, 11% from the visual condition, 60% from the condition-independent parameter and 7% from the interaction among conditions (Fig. 1S-a, top). The time course of the main component projections displays that the population clearly discriminated the left and right target and the light and dark visual condition as it is represented in Fig. 1S-a (first and second row of component time course). For the *wrist orientation task*, the variance captured by wrist orientation was 11%, by visual condition was 15%, and finally the 67% and 7% were captured by the condition-independent and the condition interaction parameters, respectively (Fig. 1S-b, top). The time course of the main component projections shows significant tuning for the two wrist orientations and the two visual conditions tested (Fig. 1S-b, first and second row of component time course). In the *grip type task*, the amount of variance captured by the grip type parameter was 17%, and that captured by visual condition was 23%. The condition-independent parameter captured the 50% of the total variance and the condition-interaction captured the 10%, as shown in Fig. 1S-c (top). Also in this case, the time course of the component projections revealed the ability of the population in discriminating between the two grip types and the two visual conditions (Fig. 1S-c, first and second row of component time course). Fig. 2S summarizes the comparison of percentages of variance captured in the three tasks we analysed. The distribution of variance corresponding to target position was significantly higher than those of wrist orientation (22%, *reach direction task vs* 11%, *wrist orientation task,* chi-squared, $P < 0.05$) and not significantly different from grip type (22%, *reach direction task* vs 17% *grip type task,* chi-squared, $P > 0.05$). The grip type parameter was significantly higher than the wrist orientation one (17%, *grip type task* vs 11% *wrist orientation task,* chi-squared, $P < 0.05$). The distribution of the task variables within each task displayed significant differences only in the *reach direction task* (11%, visual condition vs 22%, target position, chi-squared, $P < 0.05$). The amount of variance relative to the visual conditions in the *grip type task* was statistically higher than the amount of variance captured by visual condition in the *wrist orientation task* (15%, visual condition in *wrist orientation task* vs 23%, visual condition in *grip type task*, chi-squared, $P < 0.05$). Overall, the distribution of variance captured by the different task parameters is similar to that obtained by dPCA performed in the main text (see Fig. 5) where all the available task conditions were considered.

Supplemental Figures

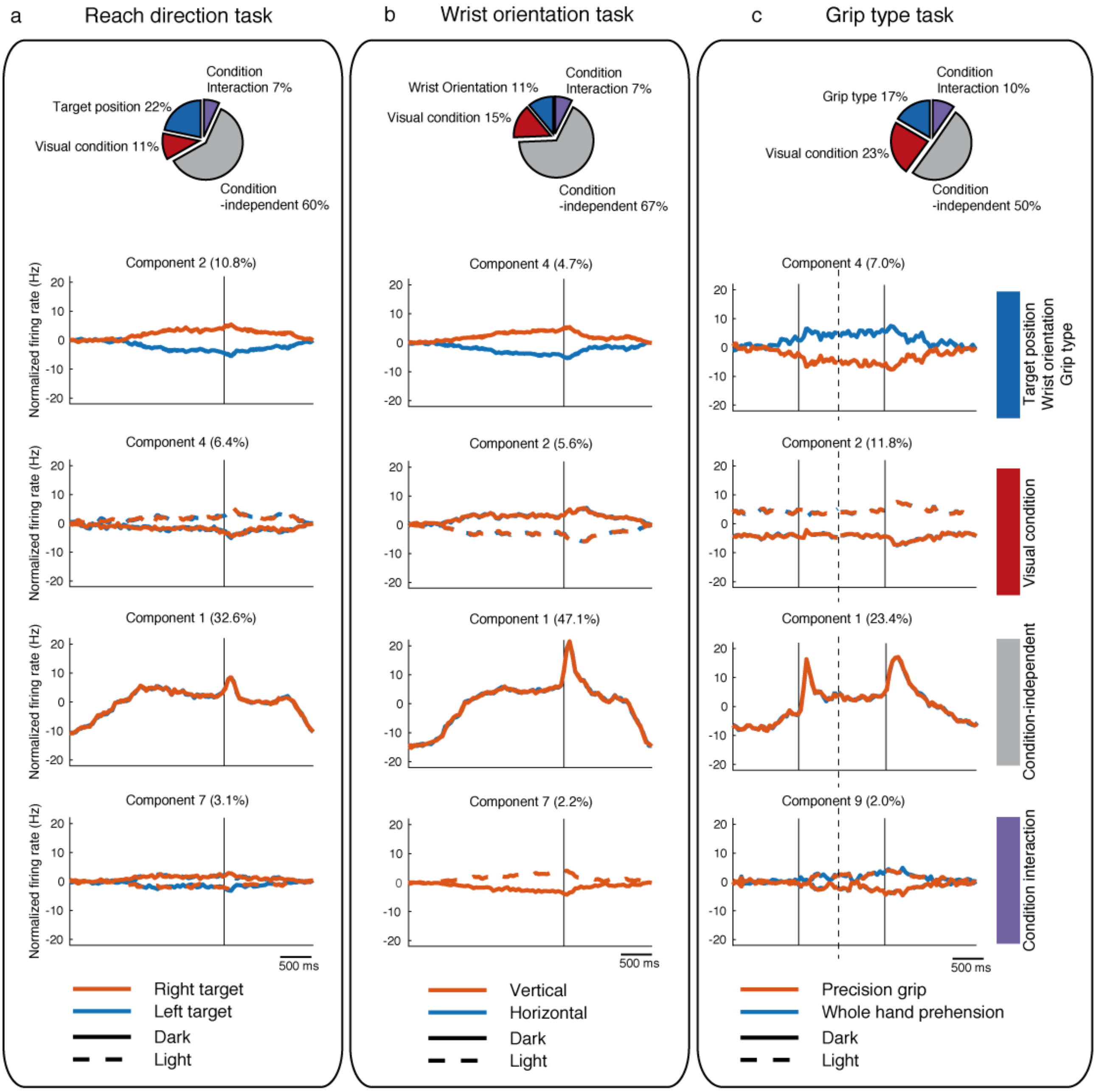

Figure S1: Demixed principal component of the three tasks with only 2 conditions per task. **a**, *reach direction task*. Top, Variance of the individual demixed principal components. Each bar shows the proportion of total variance. Pie chart shows how the total signal variance is split between parameters (visual condition and target position). Bottom, time course of the projections of single components aligned on movement onset. From top to bottom, each panel of projections of main component is relative to: target position, visual condition, condition-independent and condition interaction. **b**, *wrist orientation task*. Top, Variance of the individual demixed principal components. Each bar shows the proportion of total variance. Pie chart shows how the total variance is split between parameters (visual condition and wrist orientation). Bottom, time course of the projections of single components aligned on movement onset. From top to bottom, each panel of projections of main component is relative to: wrist orientation, visual condition, condition-indipendent and condition interaction. **c**, *grip type task*. Top, Variance of the individual demixed principal components. Each bar shows the proportion of total variance. Pie chart shows how the total signal variance is split between parameters (visual condition and grip type). Bottom, time

course of the projections of single components aligned on object illumination and movement onset. From top to bottom, each panel of projections of main component is relative to: grip type, visual condition, condition-indipendent and condition interaction.

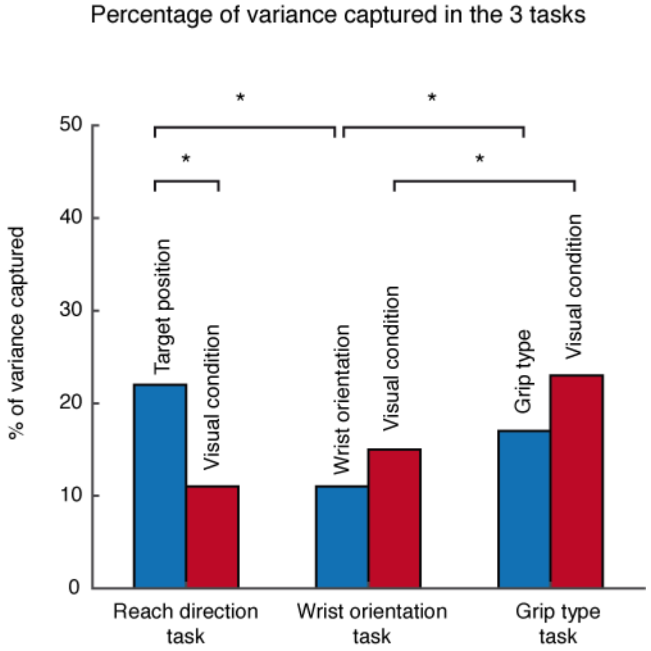

Figure S2: Distribution of variance captured in the three tasks. Blue columns show the percentage of variance captured by target position, wrist orientation and grip type parameters of the three tasks. Red columns show the percentage of variance captured by visual condition in reach direction, wrist orientation and grip type tasks, respectively. Asterisks indicate significant differences (Chi-squared, $P < 0.05$).
